# Introduction of pathogenic mutations into the mouse *Psen1* gene by Base Editor and Target-AID

**DOI:** 10.1038/s41467-018-05262-w

**Published:** 2018-07-24

**Authors:** Hiroki Sasaguri, Kenichi Nagata, Misaki Sekiguchi, Ryo Fujioka, Yukio Matsuba, Shoko Hashimoto, Kaori Sato, Deepika Kurup, Takanori Yokota, Takaomi C. Saido

**Affiliations:** 1 Laboratory for Proteolytic Neuroscience, RIKEN Center for Brain Science, Wako-shi, Saitama 351-0198 Japan; 2 Department of Neurology and Neurological Science, Graduate School of Medicine, Tokyo Medical and Dental University, Bunkyo-ku, Tokyo, 113-8510 Japan; 3 Department of Molecular and Cellular Biology, Harvard University, Cambridge, MA 02138 USA

## Abstract

Base Editor (BE) and Target-AID (activation-induced cytidine deaminase) are engineered genome-editing proteins composed of Cas9 and cytidine deaminases. These base-editing tools convert C:G base pairs to T:A at target sites. Here, we inject either BE or Target-AID mRNA together with identical single-guide RNAs (sgRNAs) into mouse zygotes, and compare the base-editing efficiencies of the two distinct tools in vivo. BE consistently show higher base-editing efficiency (10.0–62.8%) compared to that of Target-AID (3.4–29.8%). However, unexpected base substitutions and insertion/deletion formations are also more frequently observed in BE-injected mice or zygotes. We are able to generate multiple mouse lines harboring point mutations in the mouse *presenilin 1* (*Psen1*) gene by injection of BE or Target-AID. These results demonstrate that BE and Target-AID are highly useful tools to generate mice harboring pathogenic point mutations and to analyze the functional consequences of the mutations in vivo.

## Abstract (web summary)

CRISPR-guided cytidine deaminases, including BE3 (Base Editor 3) and Target-AID (activation-induced cytidine deaminase), can covert C:G base pairs to T:A at target site. Here, the authors generate missense mutations of mouse *Psen1* gene and find BE3 has higher editing efficiency than Target-AID.

## Introduction

With the rapid progress of genetic analyses using human tissues, the number of identified pathogenic mutations has increased drastically. However, significant challenges remain for the functional consequences of these mutations to be explored, not only because the number of patients with specific mutations is often small, but also because the direct evaluation of humans is impractical. In addition, clinically reported mutations are not always related to the disease^1^. Alzheimer’s disease (AD), the most common type of dementia, is no exception when it comes to confronting such challenges. Over 200 mutations in the *presenilin 1* (*PSEN1*) gene and more than 50 mutations in the *amyloid precursor protein* (*App*) gene have been identified as pathogenic familial AD (fAD) mutations (Alzforum, http://www.alzforum.org). Most of these are missense mutations with unidentified functional consequences in vivo. Introducing disease-related point mutations into research animals is one of the most reliable ways to elucidate the functional consequences of mutations and the etiology of hereditary diseases. However, generating animal models with such mutations by conventional embryonic stem cell-based gene targeting technology is time-consuming and requires considerable resources^2^. Novel gene-editing technologies such as TALEN and CRISPR/Cas9 overcome these problems, but undesired insertion/deletion (indel) frequently occurs. This is because the non-homologous end-joining pathway, in which the introduced DNA double-strand breaks (DSBs) are repaired by joining the two ends together with random indels, competes with homology-directed repair (HDR), in which the DSBs are rebuilt with template homologous DNA strands carrying the desired mutations. The efficiency of HDR is not always high enough to generate disease models^3^. Furthermore, researchers need to design template DNA for each different mutation.

Recently, novel CRISPR-Cas9-based base-editing technology has been established, in which catalytically deactivated Cas9 (dCas9) or Cas9 nickase (nCas9) is fused with cytidine deaminases to convert C:G base pairs to T:A pairs at target sites with a reduced rate of indel formation in the presence of single-guide RNAs (sgRNAs). Base Editor (BE) is a fusion protein of *Streptococcus pyogenes* Cas9 (SpCas9) and rat APOBEC1 (apolipoprotein B mRNA-editing enzyme, catalytic polypeptide-like 1)^4^, while Target-AID (activation-induced cytidine deaminase) is composed of SpCas9 and sea lamprey PmCDA1 (*Petromyzon marinus* cytosine deaminase 1)^5^. In addition to the original BE, mutant versions of BE or BE with *Staphylococcus aureus* Cas9 (SaCas9) have been generated, in which the proto-spacer adjacent motif (PAM) sequences have been changed from the NGG of the original SpCas9^6^. BE converts Cs at positions 4–8 of the sgRNA target sequences, whereas Target-AID substitutes Cs at positions 2–4, counting the PAM as positions 21–23. Both these base-editing tools have been reported to convert target bases with high efficiency in vitro^4,5^. More recently, BE has been used to introduce point mutations in mice^7,8^, zebrafish^8,9^, and human zygotes^10,11^. Although Target-AID has been shown to induce mutations in crop plants^12^, it remains unclear whether it works in mammals.

Here we introduced either BE or Target-AID mRNA together with identical sgRNAs that targeted the *Psen1* or *App* gene into mouse zygotes. Multiple animal models were generated with a number of distinct disease-related and disease-unrelated point mutations, thereby enabling the functional consequences of these mutations to be evaluated in vivo.

## Results

### Editing efficiency of BE and Target-AID in mouse zygotes

**Fig. 1:**
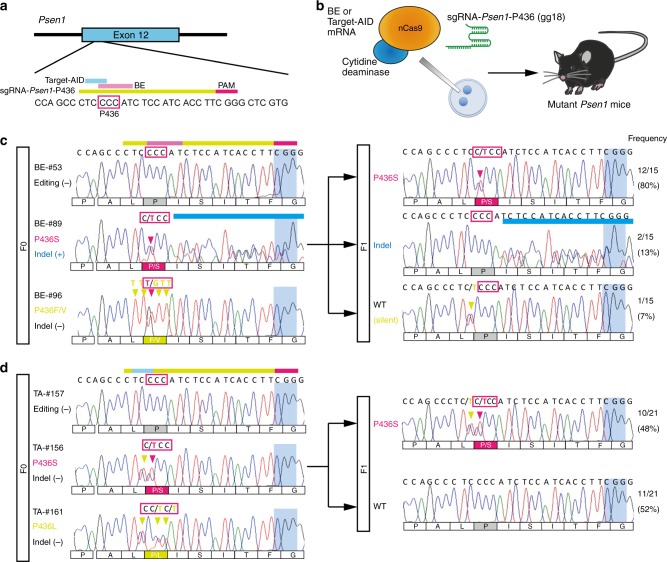
Generation of *Psen1*-P436S mice by BE and Target-AID. **a** Design of sgRNA that targets *Psen1*-P436. The activity windows of BE (pink) and Target-AID (light blue) are indicated. **b** Schematic representation of the injection of mouse zygotes for the generation of *Psen1*-P436S mice. BE or Target-AID mRNA was injected together with sgRNA-*Psen1*-P436 into the cytoplasm of mouse zygotes to generate mutant *Psen1* mice. **c**, **d** Sanger sequencing chromatograms in BE-injected (**c**) and Target-AID-injected (**d**) mice. Magenta arrowheads indicate expected substitutions, while green arrowheads indicate undesired substitutions, and blue bars indicate indels. The panels on the right represent Sanger sequencing chromatograms of F1 mice generated from the #89 (BE-injected F0) mouse (**c**) or the #156 (Target-AID-injected F0) mouse (**d**). The column on the right indicates the frequency of each genotype in the F1 generation

We first took advantage of the overlap of targetable C in the 4th position of sgRNAs in BE and Target-AID to design an sgRNA that could be used with both base-editing tools. We found that an sgRNA targeting *Psen1*-P436 had a C in the 4th position (Fig. 1a). We injected sgRNA-*Psen1*-P436 (60 ng/μl) (Fig. 1a, b) together with either BE or Target-AID mRNA (200 ng/μl) into the cytoplasm of C57BL/6J mouse zygotes to generate mutant mice carrying *Psen1*-P436S, which has been identified in fAD patients^13^. We used third-generation BE, BE3, that expressed nCas9 fused with rat APOBEC1 and uracil glycosylase inhibitor (UGI)^4^ (hereafter, BE indicates BE3 unless specified otherwise), and Target-AID composed of nCas9 fused with sea lamprey PmCDA1 and UGI^5^. As expected, BE injection induced efficient conversion of C to T at the target sites (Fig. 1c, Table 1, Supplementary Fig. 1a, b). We generated 27 mutant *Psen1* mice by BE out of a total of 43 animals (62.8%); six mice harbored silent mutations (14.0%) and 21 mice had missense mutations (48.8%) (Supplementary Fig. 1e, f). However, because the 5th C was more efficiently converted to T (39.5%) than the 4th C (14.0%), this resulted in the substitution of proline (P) to leucine (L) (CTC or CTT). Thus, mice that harbored *Psen1*-P436L were generated more frequently (32.6%) than those carrying *Psen1*-P436S (4.7%). In addition, unexpected conversion of C to A or G took place not only at the target site but also outside the BE activity window, resulting in the generation of mice with mutations such as *Psen1*-P436F, *Psen1*-P436H, *Psen1*-I437M, or *Psen1*-S438F (Supplementary Fig. 1a, b, e, f), which have not been reported as fAD-related mutations. Interestingly, some mice harbored two different mutations in different alleles at P436; these included P436S/P436L (*n* = 1, 2.3%), P436L/P436F (*n* = 2, 4.7%), and P436F/P436V (*n* = 1, 2.3%) (Supplementary Fig. 1f). Furthermore, we observed indel formation in 26.9% of the BE-injected mice (Table 1); these animals were excluded from further analysis because base conversion is sometimes masked by indel signals in the sequencing analysis and because indels in the *Psen1* gene may have affected the function of PS1 in our biochemical analyses.

**Table 1:** Summary of the zygotes used and mutant mice obtained after BE or Target-AID injections with sgRNA-*Psen1*-P436. Nine BE-injected mice and two Target-AID-injected mice were excluded to calculate frequency of substitutions due to existence of indel signals

	sgRNA	No. of injected zygotes	No. of neonates (%)	Frequency of indel (%)	Frequency of substitution (%)	Frequency of desired substitution (%)
BE	*Psen1*-P436	200	52 (26.0%)	14/52 (26.9%)	27/43 (62.8%)	2/43 (4.7%)
Target-AID	*Psen1*-P436	150	36 (24.0%)	2/36 (5.6%)	4/34 (11.8%)	1/34 (2.9%)

Following Target-AID injection, 4 out of 34 mice carried mutations in the *Psen1* gene (11.8%), which resulted in *Psen1*-P436S, *Psen1*-P436L, *Psen1*-P436I, and *Psen1*-P436T mutants (*n* = 1, 2.9% for each mutation) (Fig. 1d, Supplementary Fig. 1g). Consistent with a previous report^5^, the 3rd C was most effectively converted to T in Target-AID-injected mice, resulting in silent mutations in this case (Supplementary Fig. 1c, d). The 4th C was converted to T with 2.3% efficiency and to A with 4.7% efficiency. The 5th and 6th Cs were converted to T with 2.3% efficiency, with no other mutations observed for Target-AID. Only 2 out of 36 mice (5.6%) were found to harbor indels in Target-AID-injected mice (Table 1).

As the target sequence of sgRNA-*Psen1*-P436 was completely conserved in mouse *Psen2* (chromosome 1), we observed a C to T substitution around *Psen2*-P428 or indel formation at this position with similar efficiency to *Psen1*-P436 in BE-injected mice, including the *Psen1*-P436S founder mouse (#89) (Supplementary Fig. 2a, b). We also observed substitution of 3rd C to T in the *Psen2* gene in Target-AID-injected mice at low frequency (Supplementary Fig. 2c, d). The mice with *Psen2* mutations were excluded from subsequent biochemical experiments as *Psen2* has also been reported to act as a causative gene for fAD^14^, and these mutations could have influenced our results.

Next, to assess the off-target effects of BE and Target-AID, we searched for potential off-target sites using Cas-OFFinder^15^ and COSMID^16^. We identified 29 such sites (Supplementary Table 1) and performed targeted deep sequencing in the *Psen1*-P436S founder mice generated by BE or Target-AID. It is worth noting that we did not find any off-target site in the same chromosome as that containing *Psen1* (chromosome 12). Our results revealed that the *Psen1*-P436S mouse (#89) which was generated by BE had an off-target mutation at OFF19, which is found in the intron of the *fragile histidine triad* (*Fhit*) gene (Supplementary Fig. 3a). No apparent increase in mutations at sites other than *Fhit* and *Psen2* was observed, demonstrating the accuracy of the base-editing ability of BE and Target-AID. We confirmed germline transmission of *Psen1*-P436S in F1 mice in both BE-mediated and Target-AID-mediated lines (Fig. 1c, d). In addition, the undesired mutation in *Psen2* and the off-target mutation in the *Fhit* gene could be removed in approximately half of the F1 mice that were generated by BE by backcrossing with wild-type mice (Supplementary Fig. 3b).

To further compare the base-editing efficiencies of BE and Target-AID in vivo, we designed three different sgRNAs that targeted the mouse *App* gene and injected them with either BE or Target-AID mRNA (Supplementary Fig. 4a–f). The base-editing efficiency of BE was consistently higher (10.0–53.6%) than that of Target-AID (3.4–29.8%) in each of the three targeted regions. However, unexpected substitutions or indel formation were also observed more frequently in BE-injected zygotes (Supplementary Table 2). These results indicate that BE has a high base-editing efficiency but with less precision, whereas Target-AID edits bases less efficiently but with relatively high accuracy.

### Phenotypes of BE-generated and Target-AID-generated *Psen1-*P436S mice

PS1 comprises the catalytic component of the γ-secretase complex, which cleaves type I transmembrane proteins, including APP^17^. Several mutant *Psen1* knock-in mouse lines were previously reported^18–25^, and some mice showed aborted maturation of PS1 protein and alteration in processing of its substrates. To analyze the functional consequence of *Psen1*-P436S mutation in vivo, we first evaluated the viability of homozygous *Psen1*-P436S neonates because some *Psen1* mutations cause embryonic/perinatal lethality or developmental defects when the mutations exist in a homozygous state. The homozygous mutant mice appeared viable and indistinguishable from wild-type littermates (Supplementary Fig. 5a). Western blot analysis indicated that expression levels of N-terminal and C-terminal fragments (NTFs and CTFs) of PS1 protein remained unaltered in heterozygous or homozygous mutant mice (Supplementary Fig. 5b).

**Fig. 2:**
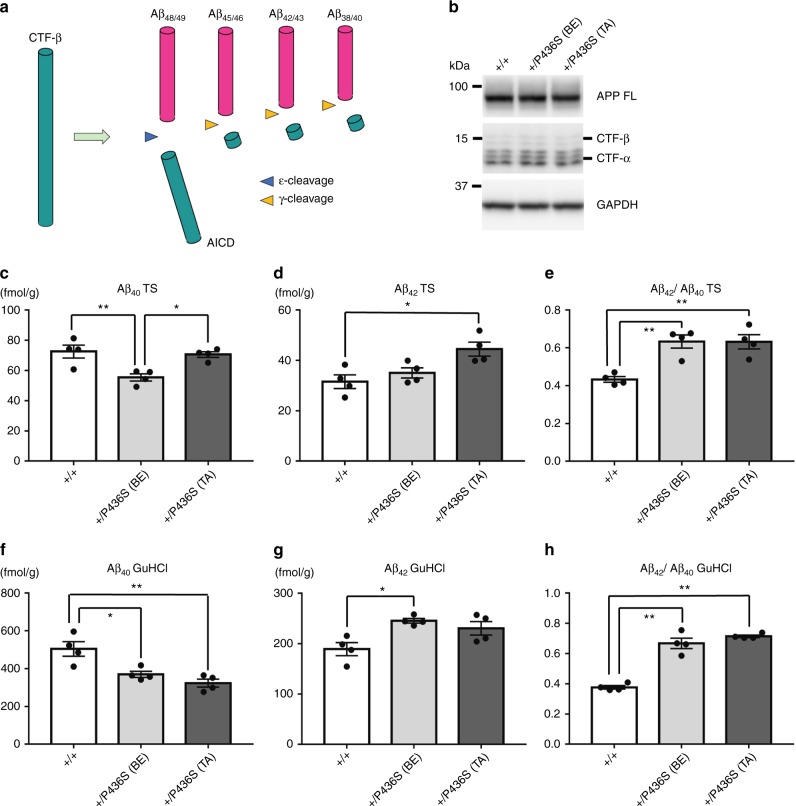
Phenotypic analysis of *Psen1*-P436S mice generated by BE and Target-AID. **a** Sequential processing of APP CTF-β by γ-secretase. CTF-β is first cleaved by an endopeptidase-like cleavage (ε-cleavage) to produce Aβ_48_ and Aβ_49_, and APP intracellular domain (AICD). Subsequently, γ-secretase cleaves Aβ_48_ and Aβ_49_ by a carboxypeptidase-like cleavage (γ-cleavage) to generate smaller Aβ species. **b** Western blot analysis of the full-length (FL) and CTFs of APP in the *Psen1*-P436S mouse brain. Western blot analysis using anti-APP FL (22C11) and CTFs (APP-CTF) was performed using brain samples of 3-month-old heterozygous *Psen1*-P436S mice generated by BE or Target-AID. The full-length images are shown in Supplementary Fig. 7. **c**–**h** Aβ ELISA of *Psen1*-P436S mouse brains. Cortices from 3-month-old heterozygous *Psen1*-P436S mice generated by BE or Target-AID and littermate wild-type mice (*n* = 4 for each group) were homogenized and fractionated into Tris-HCl-buffered saline-soluble (TS) (**c**–**e**) and guanidine-HCl-soluble (GuHCl) fractions (**f**–**h**) and subjected to ELISAs for Aβ_40_ and Aβ_42_. *P ≤ 0.05 and **P ≤ 0.01 (one-way ANOVA followed by Tukey’s post hoc analysis). WT: wild type, BE: Base Editor 3, TA: Target-AID. Data represent mean ± s.e.m. Genotypes of each mouse are shown in Supplementary Table 3. The information on the sex and age of the mice is provided in Supplementary Table 9

CTF-β, which is generated by proteolytic processing of APP by β-secretase (β-site APP cleaving enzyme 1 or BACE1), is one of the substrates of γ-secretase. γ-Secretase first cleaves CTF-β by an endopeptidase-like cleavage (ε-cleavage) to produce long forms of β-amyloid (Aβ) peptide, Aβ_48_ and Aβ_49_ (Fig. 2a)^26^. Subsequently, γ-secretase trims these long Aβs by a carboxypeptidase-like cleavage (γ-cleavage) to generate smaller Aβ species of various lengths. The profile of Aβ peptide production is reported to be altered in fAD patients: Aβ_40_, which is composed of 40 amino acids, is the predominant species in healthy subjects and tends not to aggregate. In contrast, longer Aβs such as Aβ_42_ and Aβ_43_, which exhibit higher pathogenicity and are prone to aggregation, are increased in the fAD brain^27^. Western blot analysis revealed that full-length APP and CTF-β were not altered in the heterozygous *Psen1*-P436S mice (Fig. 2b, Supplementary Figs. 6, 7). Next, we performed enzyme-linked immunosorbent assay (ELISA) for Aβ_40_ and Aβ_42_ in heterozygous *Psen1*-P436S mouse brains (Fig. 2c–h). Aβ_40_ was decreased and Aβ_42_ was increased in the guanidine hydrochloride (GuHCl)-soluble fractions of *Psen1*-P436S lines generated by BE or Target-AID (Fig. 2f–h), resulting in an approximately 1.7-fold increase in the Aβ_42_/Aβ_40_ ratio. This increased Aβ_42_/Aβ_40_ ratio was reproduced in the Tris-HCl-buffered saline-soluble fraction of the mutant lines (Fig. 2c–e). These results suggest that introduction of *Psen1*-P436S in mice alters the carboxypeptidase-like cleavage (γ-cleavage) activity of γ-secretase. No age-dependent increase of Aβ (Supplementary Fig. 5c–f) nor amyloid depositions (Supplementary Fig. 5g) in the brain was observed in *Psen1*-P436S mice. Although these results are consistent with previously reported *Psen1* mutation knock-in mice^19,21,24,25^, further characterization is required to demonstrate the validity of utilizing *Psen1*-P436S mice for AD research. Interestingly, the biochemical profile of Aβ production in the *Psen1*-P436L founder mice that harbored P436L alleles at 35–52% by mosaicism was similar to that of the *Psen1*-P436S animals (Supplementary Fig. 8), indicating that the fAD-unrelated mutation had a similar effect to *Psen1*-P436S on PS1 functions. These results demonstrate that the BE-mediated and Target-AID-mediated generation of mutant mice is an effective way to analyze the functional consequences of disease-related and disease-unrelated point mutations in vivo.

### Efficient generation of multiple mutant mouse lines by BE

**Fig. 3:**
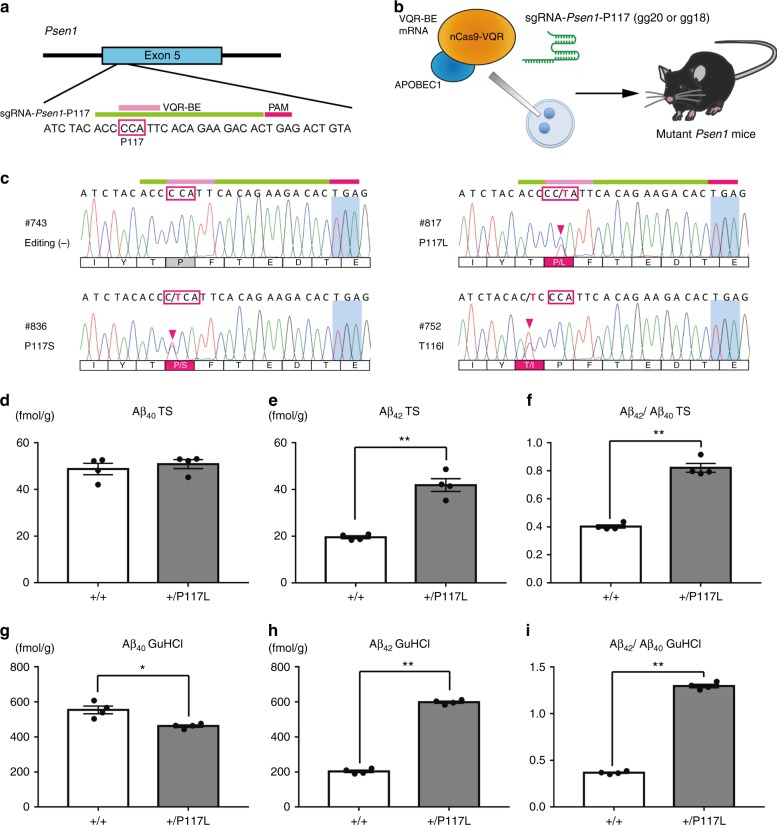
Generation of mutant *Psen1*-P117 mice by VQR-BE. **a** Design of sgRNA that targets *Psen1*-P117. The target region of VQR-BE (pink) is indicated. **b** Schematic representation of the injection of mouse zygotes for the generation of mutant *Psen1*-P117 mice. VQR-BE mRNA was injected together with sgRNA-*Psen1*-P117 into the cytoplasm of mouse zygotes to generate mutant *Psen1* mice. **c** Sanger sequencing chromatograms of VQR-BE-injected mice. Magenta arrowheads indicate substitutions that result in fAD-related mutations. **d**–**i** Aβ ELISA of *Psen1*-P117L mouse brains. Cortices from 3-month-old heterozygous *Psen1*-P117L mice generated by VQR-BE and littermate wild-type mice (*n* = 4 for each group) were homogenized and fractionated into Tris-HCl-buffered saline-soluble (TS) and guanidine-HCl-soluble (GuHCl) fractions and subjected to ELISAs for Aβ_40_ and Aβ_42_. *P ≤ 0.05 and **P ≤ 0.01 (Student’s two-tailed *t*-test). Data represent mean ± s.e.m. Genotypes of each mouse are shown in Supplementary Table 3. The information on the sex and age of the mice is provided in Supplementary Table 9

Next, we capitalized on the flexible character of BE, which can edit multiple bases with variable patterns, and sought to generate multiple mouse lines that harbor different point mutations. We found that *Psen1*-P117 is a suitable target for this purpose, because the original CCA (P) can be converted to TCA (S), CTA (L), or TTA (L), and because both *Psen1*-P117S and *Psen1*-P117L have been reported to be pathogenic fAD mutations (Fig. 3a). To target *Psen1*-P117, we used a VQR (D1135V, R1335Q, and T1337R)-BE variant in which the PAM sequence is changed from NGG to NGA^6^. We injected sgRNA-*Psen1*-P117 (30 ng/μl) together with VQR-BE mRNA (200 ng/μl) into the cytoplasm of C57BL/6J zygotes (Fig. 3b). In this series, we prepared two sgRNAs of different length, as the length could affect the base-editing efficiency in a manner similar to that of other modified CRISPR/Cas9 tools^28^. SgRNA (gg20) has a 20-base target sequence with two additional mismatched Gs upstream of the target; sgRNA (gg18) has 18 bases of target sequence with two additional mismatched Gs. Sequencing analysis in these mice (*n* = 77) revealed that the targeted Cs were efficiently substituted to T. Consequently, we obtained *Psen1*-P117S (*n* = 12, 15.6%) and *Psen1*-P117L (*n* = 27, 35.1%) mice at high frequency (Fig. 3c, Table 2). In addition, unexpected substitutions resulted in the generation of *Psen1*-T116I (*n* = 1, 1.3%), *Psen1*-T116N (*n* = 2, 2.6%), *Psen1*-P117A (*n* = 2, 3.9%), and *Psen1*-P117R (*n *= 1, 1.3%) mutations, which also cause fAD (Supplementary Fig. 9). We also obtained mice harboring fAD-unrelated mutations, such as *Psen1*-P117Q (*n* = 8, 10.4%) and *Psen1*-P117T (*n* = 3, 3.9%) (Supplementary Fig. 9). The base-editing efficiency was 80.0% with sgRNA (gg20) and 37.8% with sgRNA (gg18), while indel formation was 25.9% with sgRNA (gg20) and 16.3% with sgRNA (gg18) (Table 2). When we performed ELISA for Aβ using brain samples from the heterozygous *Psen1*-P117L mice, we found that they showed increases in Aβ_42_ and in the Aβ_42_/Aβ_40_ ratio, both in the Tris-HCl-buffered saline-soluble fraction and GuHCl-soluble fraction (Fig. 3d–i). Western blot analysis revealed that full-length APP and CTF-β were not altered in these heterozygous *Psen1*-P117L mice (Supplementary Fig. 6), suggesting that the P117L mutation in the PS1 protein causes alteration of carboxypeptidase-like cleavage (γ-cleavage) activity of γ-secretase, similar to that of P436S.

**Table 2:** Summary of the zygotes used and mutant mice obtained after VQR-BE injections with sgRNA-*Psen1*-P117. Fourteen sgRNA (gg20)-injected mice and six sgRNA (gg18) injected mice were excluded to calculate frequency of substitutions due to existence of indel signals

	sgRNA	No. of injected zygotes	No. of neonates (%)	Frequency of indel (%)	Frequency of substitution (%)	Frequency of expected substitution (%)
VQR-BE	*Psen1*-P117 (gg20)	150	54 (36.0%)	14/54 (25.9%)	32/40 (80.0%)	25/40 (62.5%)
	*Psen1*-P117 (gg18)	150	43 (28.7%)	7/43 (16.3%)	14/37 (37.8%)	10/37 (27.0%)

## Discussion

In our experiments, BE and Target-AID were able to introduce point mutations with remarkably high efficiency in vivo compared to conventional gene-targeting or CRISPR/Cas9-based knock-in technologies. Importantly, our mutant *Psen1* mice generated by BE or Target-AID recapitulated the features of previously reported mutant *Psen1* knock-in mice, suggesting that these base-editing tools could be highly useful to analyze the functional consequences of the introduced mutations in vivo. In addition to the high editing efficiencies, these base-editing tools have several advantages over conventional methods; no requirement of donor DNAs and introduction of silent mutations to prevent sequential cleavage by Cas9, and simultaneous generation of multiple mutant lines, especially in the cases where BE is used. Although there are some limitations to using base editing, recent advances in this field are solving some of these issues: evolved versions of the tools dramatically increase the number of targetable regions^29,30^, and recently established adenine BE, which can convert A:T pairs to G:C pairs, partially cancels the restrictions for the requirement of the PAM sequence^29^. Base-editing tools should thus facilitate exploration of the unidentified functional consequences of each pathogenic mutation.

In our study, BE unexpectedly induced indel formation in mouse zygotes at high frequency (6.7–35.6%, Table 1, 2, Supplementary Table 2) compared to the previous in vitro studies (average 1.1%^4^). The reason for this discrepancy is unclear; however, Kim et al. also reported indel formation in mice generated by BE at a similar frequency to ours (one out of nine *Dmd*-targeted mice (11.1%) and two out of seven *Tyr*-targeted mice (28.6%))^7^. The genome-editing or DNA repair mechanism in vivo might be different from those present in cultured cells. BE4, the next generation after BE3 that has an additional UGI in its C-terminus, is reported to induce more precise base conversion compared to BE3^31^. It is therefore possible that base excision repair initiated by uracil DNA glycosirase may be responsible for causing DSB and subsequent indel formation.

The base-editing efficiency of Target-AID in mouse zygotes was lower than that of BE, not only at the 4th C but also at the 3rd C in some cases, the point at which Target-AID could most effectively edit bases (Supplementary Figs. 1, 4). As Target-AID uses sea lamprey PmCDA1 to convert the target bases, the most appropriate temperature for its application is likely to be around 25 °C^5^, which might explain the low base-editing efficiency in mouse embryos in the present study, which was conducted at 37 °C. Although the base-editing efficiency of Target-AID was lower than that of BE in mice, the frequency of undesired conversion and indel formation was also lower. In addition to targeting the 2nd and 3rd Cs in sgRNA sequences, Target-AID might also be a better option than BE for introducing precise point mutations targeting the 4th C.

Because mouse Aβ has low amyloidogenic potential that might be caused by the existence of three different amino acids compared to human Aβ^18,32–34^, it is difficult to recapitulate full features of AD such as Aβ plaque formation or behavioral alterations in mice with a single *Psen1* mutation. However, it is noteworthy that our *Psen1*-P436S and *Psen1*-P117L mice showed alteration of their Aβ profiles, which is one of the important pathological features of AD, in a heterozygous state similar to human fAD patients. To further characterize our mice for AD research, they need to be crossed with mice harboring humanized sequences of Aβ peptide, such as our *App* knock-in mice^35^.

In addition to *Psen1*-P436S and *Psen1*-P117L, we identified *Psen1*-P436L as a potential novel pathogenic mutation that has not been previously reported. Interestingly, the Iberian mutation in *App* (*App*-I716F) was experimentally predicted by Beyreuther and colleagues to be pathogenic many years before it was actually identified as a clinical mutation^36,37^. Screening using base-editing technology could therefore prove valuable for the identification of such unknown pathogenic mutations or protective variants.

## Methods

### Animals

All animal experiments were carried out in accordance with the RIKEN Center for Brain Science guidelines for animal experimentation. C57BL/6J and ICR mouse strains were used as embryo donors and foster mothers, respectively. Wild-type C57BL/6J mice were used when backcrossing the mutant *Psen1* mice. The information on the sex and age of the mice is provided in Supplementary Table 9.

### In vitro transcription

pCMV-BE3 (Addgene plasmid #73021), pBK-VQR-BE3 (Addgene plasmid #85171), and pcDNA3.1_pCMV-nCas-PmCDA1-ugi pH1-gRNA (Addgene plasmid #79620) were obtained from Addgene. pCMV-BE3 and pBK-VQR-BE3 were gifts from David Liu. pcDNA3.1_pCMV-nCas-PmCDA1-ugi pH1-gRNA (HPRT) (Addgene plasmid #79620) was a gift from Akihiko Kondo. The mRNA template for BE, VQR-BE, and Target-AID were prepared by PCR using Herculase II Fusion DNA Polymerase (Agilent Technologies, #600675) with primers as listed in Supplementary Table 4. BE, VQR-BE, and Target-AID mRNAs were synthesized using the mMESSAGE mMACHINE T7 Ultra Transcription Kit (Thermo Fisher Scientific, AM1345). Templates for in vitro transcription of sgRNAs against each of the *Psen1* mutations and the *App* gene were prepared by PCR using Herculase II Fusion DNA Polymerase with primers as listed in Supplementary Table 5. The sgRNAs were synthesized using the MEGAshortscript T7 Transcription Kit (Thermo Fisher Scientific, AM1354). Transcribed mRNAs and sgRNAs were purified using the MEGAclear Transcription Clean-Up Kit (Thermo Fisher Scientific, AM1908).

### Microinjection into mouse zygotes

RNA solutions containing BE, VQR-BE, or Target-AID mRNA (200 ng/μl), and sgRNAs (60 ng/μl for *Psen1*-P436, and 30 ng/μl for *Psen1*-P117) were injected into the cytoplasm of C57BL/6J zygotes. After incubation at 37 °C for 1 day, embryos at the 2-cell-stage were transferred to host ICR female mice.

### Genotyping

Genomic DNA was extracted from mouse tails. The tail samples were lysed in lysis buffer (10 mM Tris-HCl (pH 8.5), 5 mM EDTA (pH 8.0), 0.2% SDS, 200 mM NaCl, 20 µg/ml proteinase K), and extracted genomic DNA was directed to PCR and subsequent Sanger sequencing using the primers listed in Supplementary Table 6. For the mice harboring multiple mutations, TA cloning using TA PCR Cloning Kit (BioDynamics Laboratory Inc., pTAC-1), single-colony PCR, and Sanger sequencing using M13-BDFw and M13-BDRev primers (Supplementary Table 6) were performed.

### Targeted deep sequencing

Off-target sites that accepted up to three mismatches were determined by Cas-OFFinder (http://www.rgenome.net/cas-offinder/)^15^ and COSMID (https://crispr.bme.gatech.edu/)^16^. Target sites were amplified from tail genomic DNA by PCR using Herculase II Fusion DNA Polymerase with the primers listed in Supplementary Tables 7 and 8. The targeted deep sequencing was performed using an Illumina Miseq system.

### Enzyme-linked immunosorbent assay

Mouse cortices were homogenized in 50 mM Tris-HCl (pH 7.6) containing 150 mM NaCl (TBS) and the protease inhibitor cocktail complete mini (Roche Diagnostics) with a Teflon-glass homogenizer. After centrifugation at 200,000 × *g* for 20 min at 4 °C, the supernatant was collected (TS fraction). The pellet was dissolved in 6 M guanidine-HCl buffer. The solubilized pellet was centrifuged at 200,000 × *g* for 20 min at room temperature and then used as the insoluble fraction (GuHCl fraction). The amounts of Aβ_40_ and Aβ_42_ in each fraction were determined by Aβ ELISA kit (Wako, 294-62501 and 294-62601), according to the manufacturer’s instructions^38^.

### Western blot analysis

Homogenates from brain cortices at postnatal day 5 and 3-month-old mice were subjected to sodium dodecyl sulfate-polyacrylamide gel electrophoresis (SDS-PAGE), and transferred electrophoretically to a 0.22-μm PVDF membrane. Antibodies against APP (22C11, Millipore, #MAB348, 1:2000), APP-CTF (Sigma, #A8717, 1:10,000), GAPDH (TREVIGEN, #2275-PC-100, 1:5000), PS1-NTF and PS1-CTF (1:2000, kind gifts from Dr. Tomita of Tokyo University) were used^39,40^. Immunoreactive bands on the membrane were visualized with ECL Select (GE Healthcare) and scanned with a LAS-3000mini Lumino Image analyzer (Fujifilm).

### Immunohistochemical analysis

Immunohistochemical staining against Aβ (N1D, 1:200) was performed in 9-month-old *Psen1*-P436S mice and 24-month-old NL-F mice. After deparaffinization of paraffin-embedded mouse brain sections, antigen retrieval was performed by autoclave treatment (121 °C for 5 min). The sections were rinsed several times, blocked for 30 min, and then incubated with primary antibody in blocking solution overnight. A biotinylated secondary antibody and tyramide signal amplification (PerkinElmer) were used for detection of amyloid pathology. Photographic data were captured using a NanoZoomer Digital Pathology C9600 (Hamamatsu Photonics).

### Statistical analyses

All data are shown as the mean ± s.e.m. For comparison between two groups, data were analyzed by Student’s *t*-test. For comparisons among three groups, we used one-way analysis of variance followed by Tukey’s post hoc analysis. The data were collected and processed in a randomized and blinded manner.

### Data availability

Sequencing information has been uploaded to DNA Data Bank of Japan (DDBJ) Sequencing Read Archive (DRA) with the accession code DRA007028. The data related to the findings of this study are available from the corresponding authors on request.

## Electronic supplementary material

Supplementary Information
