# Supplementary material for: Introduction of pathogenic mutations into the mouse Psen1 gene by Base Editor and Target-AID
Source: Nat Commun. 2018 Jul 24;9:2892. doi: 10.1038/s41467-018-05262-w (PMC6057936; doi:10.1038/s41467-018-05262-w)
Supplement: Supplementary file 1 — Supplementary Information [file 41467_2018_5262_MOESM1_ESM.pdf]

Supplementary Information

Introduction of pathogenic mutations into the mouse *Psen1* gene by Base Editor and Target-AID

Sasaguri *et al.*

a

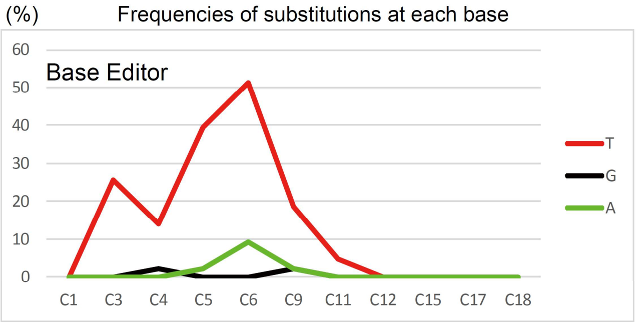

b

Base Editor

Position	1	2	3	4	5	6	7	8	9	10	11	12
Base	C	T	C	C	C	C	A	T	C	T	C	C
Substitution	-	-	T	T / G	T / A	T / A	-	-	T / G / A	-	T	-
Frequency (%)	0	0	25.6	14.0 / 2.3	39.5 / 2.3	51.2 / 9.3	0	0	18.6/2.3/2.3	0	4.7	0
Amino acid #	435			436			437			438		
Amino acid	L			P > S, L, F, H, V			I > M			S > F		

c

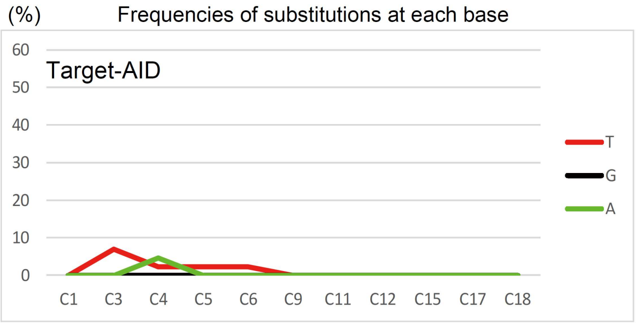

d

Target-AID

Position	1	2	3	4	5	6	7	8	9	10	11	12
Base	C	T	C	C	C	C	A	T	C	T	C	C
Substitution	-	-	T	T / A	T	T	-	-	-	-	-	-
Frequency (%)	0	0	7.0	2.3 / 4.7	2.3	2.3	0	0	0	0	0	0
Amino acid #	435			436			437			438		
Amino acid	L			P > S, L, T, I			I			S		

e

Base Editor

Single mutation	
P436S	1 (2.3%)
P436L	11 (25.6%)
P436F	1 (2.3%)
P436H	1 (2.3%)
I437M	1 (2.3%)
S438F	1 (2.3%)
Total	16 /43 (37.2%)

f

Base Editor

Multiple mutations	
P436S / L	1 (2.3%)
P436L / F	2 (4.7%)
P436F / V	1 (2.3%)
P436L + S438F	1 (2.3%)
Totai	5 / 43 (11.6%)

g

Target-AID

Single mutation	
P436S	1 (2.9%)
P436L	1 (2.9%)
P436T	1 (2.9%)
P436I	1 (2.9%)
Total	4 / 34 (11.8%)

Supplementary figure 1. Summary of base editing and generation of mutant *Psen1* mice after BE and Target-AID injections targeting *Psen1*-P436.

(a,b) Base editing efficiency of BE at each base in the target site. **(c,d)** Base editing efficiency of Target-AID at each base in the target site. **(e,f)** Summary of number of mutant *Psen1* mice obtained after BE injection. Some mice harbored multiple mutations in a same allele (P436L + S438F) or different alleles (P436S/L, P436L/F, and P436F/V). **(g)** Summary of number of mutant *Psen1* mice obtained after Target-AID injection.

a

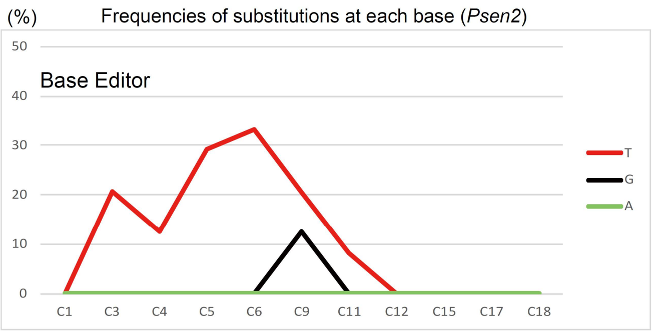

b

Base Editor

1	2	3	4	5	6	7	8	9	10	11	12	13	14	15	16	17	18	19	20
C	T	C	C	C	C	A	T	C	T	C	C	A	T	C	A	C	C	T	T
–	–	T	T	T	T	–	–	T/G	–	T	–	–	–	–	–	–	–	–	–
0	0	20.8	12.5	29.2	33.3	0	0	20.5/12.5	0	8.3	0	0	0	0	0	0	0	0	0
	427			428			429			430			431			432		433	
	L			P > S,L,F			I > M			S > F			I			T		F	

c

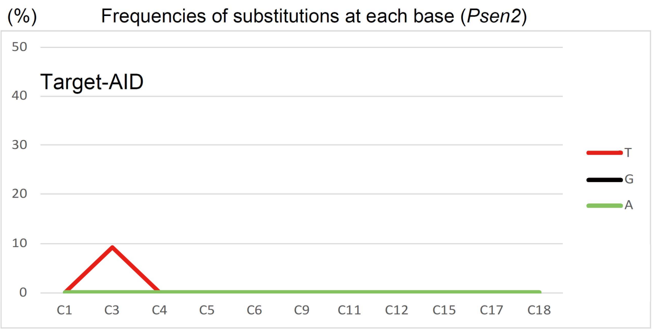

d

Target-AID

1	2	3	4	5	6	7	8	9	10	11	12	13	14	15	16	17	18	19	20
C	T	C	C	C	C	A	T	C	T	C	C	A	T	C	A	C	C	T	T
–	–	T	–	–	–	–	–	–	–	–	–	–	–	–	–	–	–	–	–
0	0	9.1	0	0	0	0	0	0	0	0	0	0	0	0	0	0	0	0	0
	427			428			429			430			431			432		433	
	L			P			I			S			I			T		F	

Supplementary figure 2. Summary of base editing in the *Psen2* gene in BE-injected mice targeting *Psen1*-P436.

Base editing efficiency of BE (a,b) and Target-AID (c,d) at each base in the *Psen2* gene. The base editing efficiency was similar to that in the *Psen1* gene as the sgRNA target sequence was completely conserved in *Psen2*. Some mice had P428L, P428S, P428F, I429M, or S430F mutations in the *Psen2* gene in mice generated by BE whereas no mouse had amino acid substitution in mice generated by Target-AID.

a

b

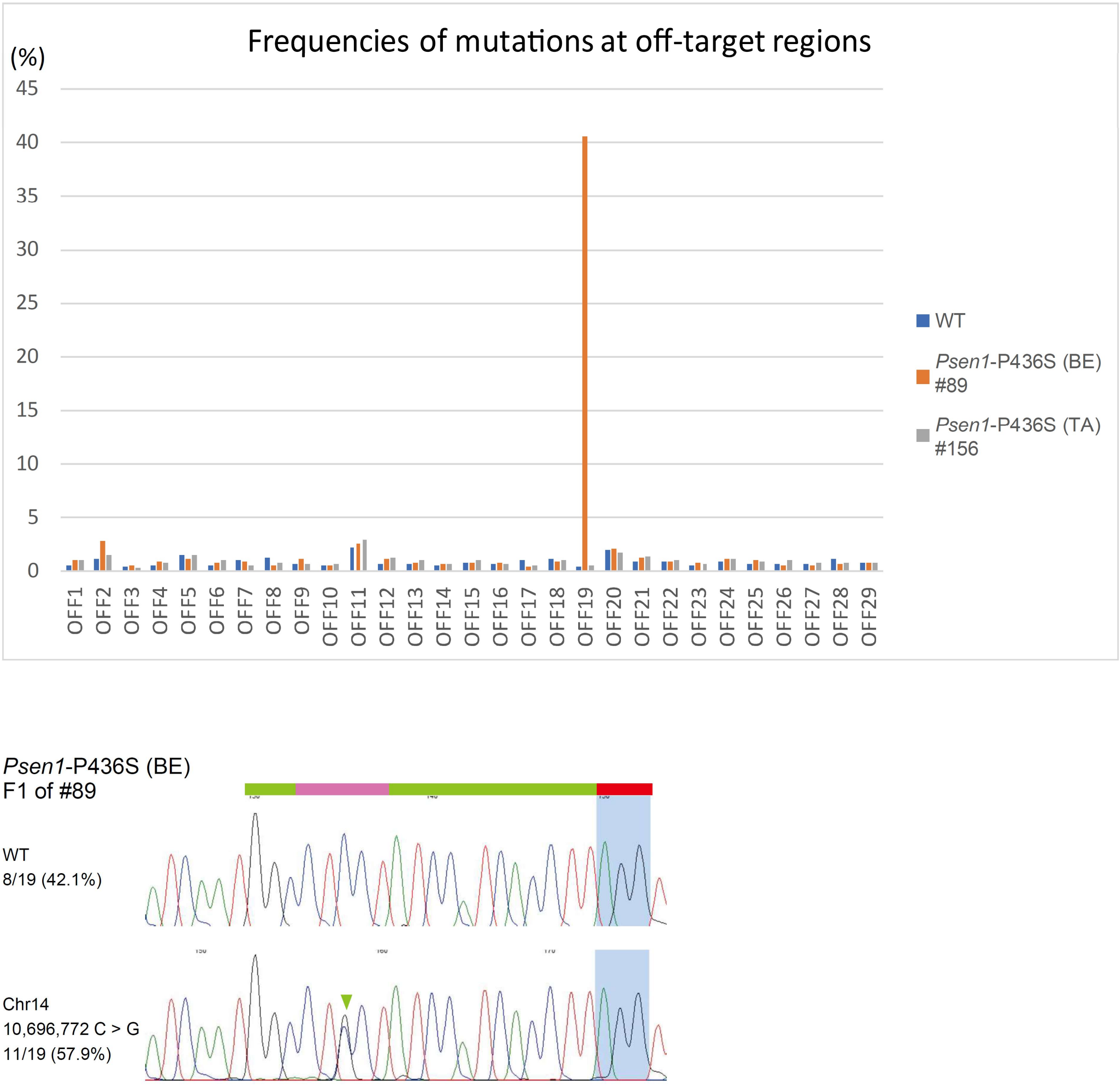

Supplementary figure 3. Off-target mutations in *Psen1*-P436S mice generated by BE or Target-AID. (**a**) Frequencies of mutations at predicted off-target sites. Potential off-target sites were searched by Cas-OFFinder and COSMID (see methods, the targets are listed in Supplementary table 1). PCR amplicons at each region were directed to targeted deep sequencing using an Illumina MiSeq. (**b**) Sanger sequencing chromatograms in *Psen1*-P436S F1 mice generated by BE. As expected, the off-target mutation could be removed by backcrossing with wild-type C57BL/6J mice.

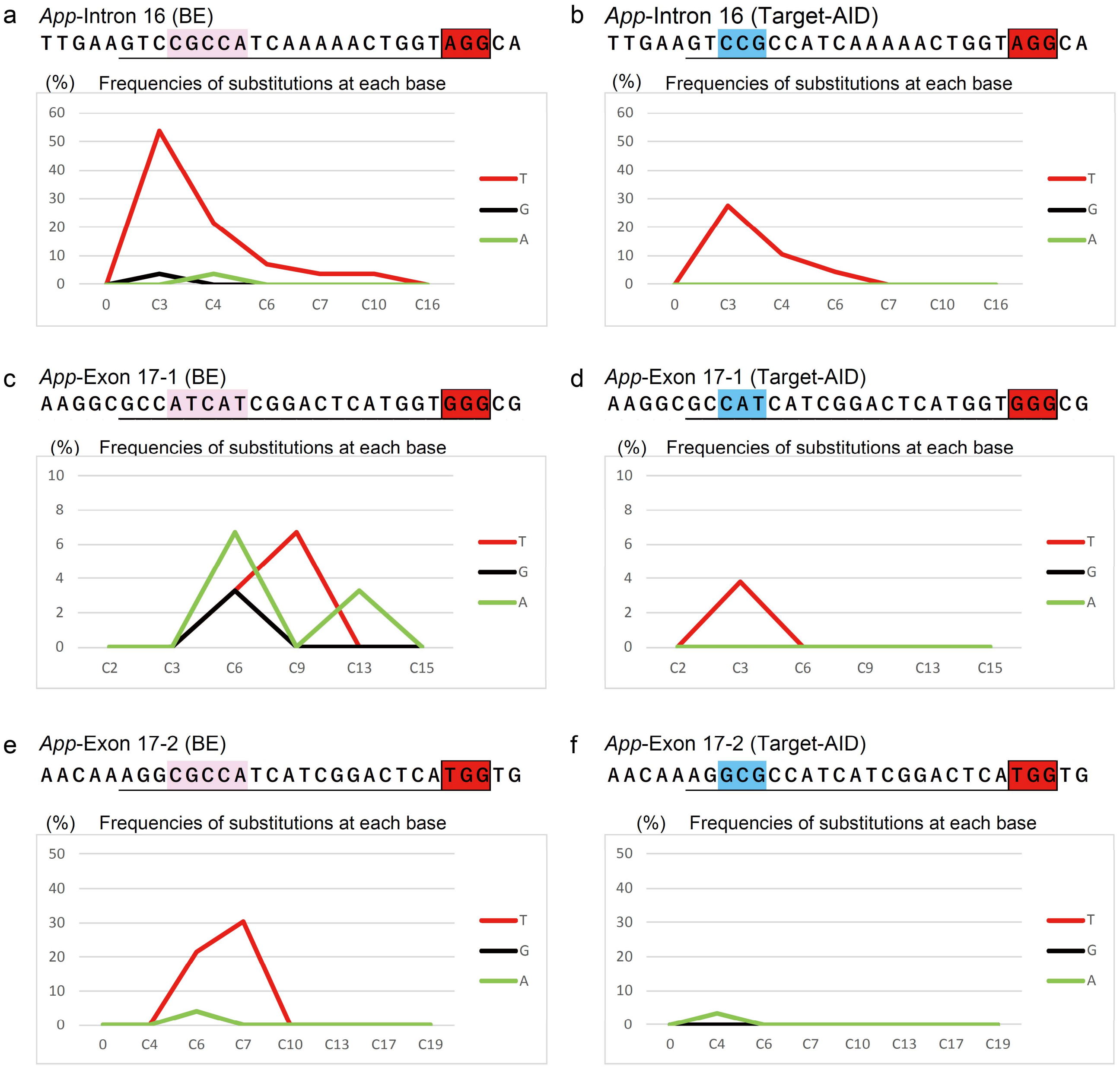

Supplementary figure 4. Summary of base editing in mouse zygotes after BE and Target-AID injections targeting the *App* gene.

(a-f) Base editing efficiency of BE or Target-AID at each base in the *App* gene. Identical sgRNAs were used either with BE (a, c, e) or Target-AID (b, d, f) to target Cs in the *App* gene.

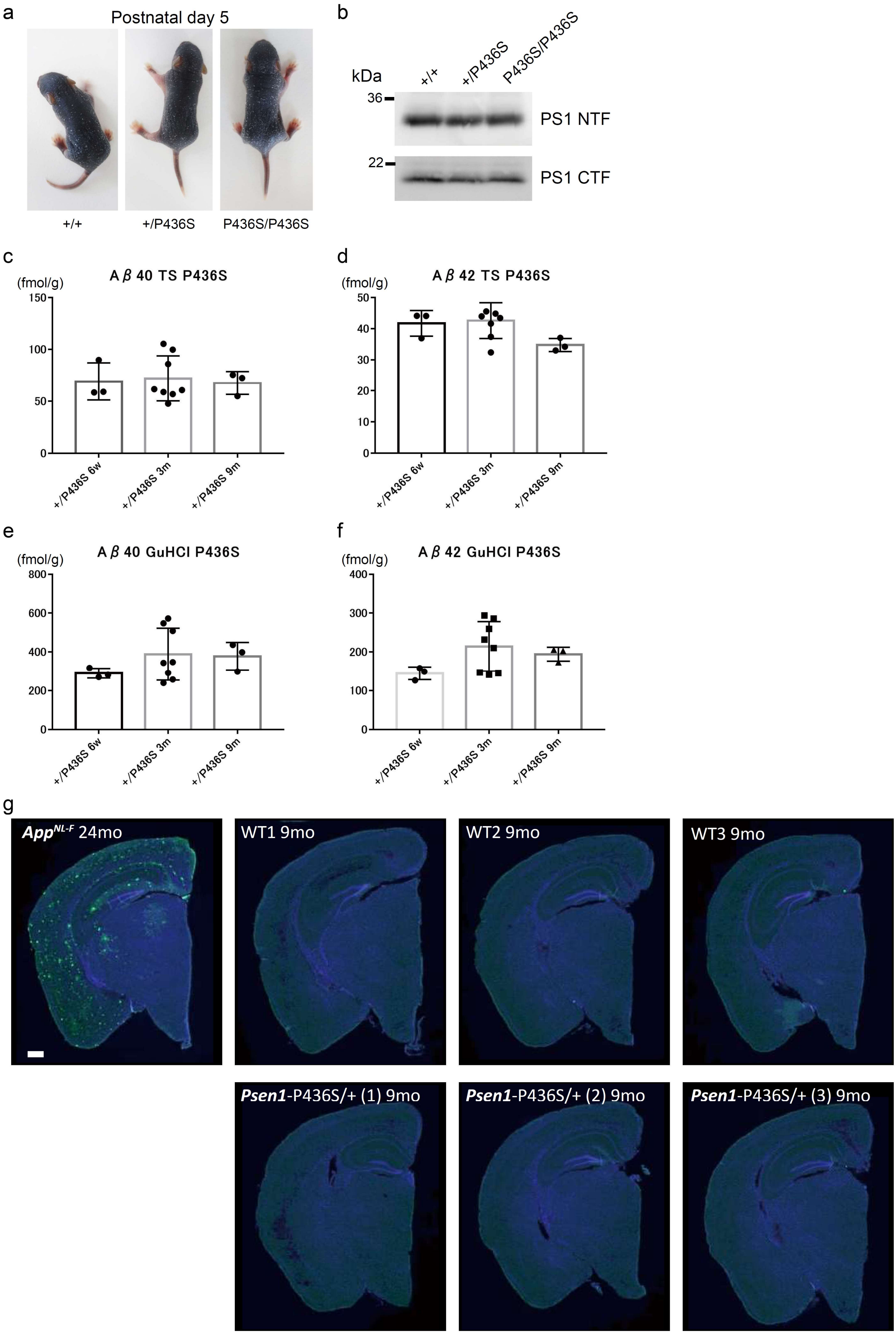
a
Postnatal day 5
+/+
+/P436S
P436S/P436S
b
kDa
+/+
+/P436S
P436S/P436S
36
22
PS1 NTF
PS1 CTF
c
Aβ 40 TS P436S
(fmol/g)
+/P436S 6w
+/P436S 3m
+/P436S 9m
d
Aβ 42 TS P436S
(fmol/g)
e
Aβ 40 GuHCl P436S
(fmol/g)
f
Aβ 42 GuHCl P436S
(fmol/g)
g
App^NL-F 24mo
WT1 9mo
WT2 9mo
WT3 9mo
Psen1-P436S/+ (1) 9mo
Psen1-P436S/+ (2) 9mo
Psen1-P436S/+ (3) 9mo

Supplementary figure 5. Characterization of *Psen1*-P436S mice generated by BE or Target-AID.

(**a**) Appearance of wild-type, heterozygous and homozygous *Psen1*-P436S mice at postnatal day 5. (b) N-terminal fragments (NTF) and C-terminal fragments (CTF) of PS1 in the brain of wild-type, heterozygous and homozygous *Psen1*-P436S mice (postnatal day 5) were detected by anti-PS1 antibodies (kind gifts from Dr. Tomita of Tokyo University). (**c-f**) $A\beta_{40}$ and $A\beta_{42}$ ELISA in the heterozygous *Psen1*-P436S mouse brain at different age (6-week-old, n=3; 3-month-old, n=8; and 9-month-old, n=3). Cortices from heterozygous *Psen1*-P436S mice generated were homogenized and fractionated into Tris-HCl-buffered saline (TS) (**c, d**) and guanidine-HCl-soluble (GuHCl) (**e, f**) fractions and subjected to the ELISAs for Aβ40 and Aβ42. Data represent mean ± s.e.m. (**g**) Immunohistochemistry using Aβ antibody (N1D) in heterozygous *Psen1*-P436S mice (9 months old, n=3). Amyloid deposition was not detected in heterozygous *Psen1*-P436S mice (a 24-month-old $App^{NL\text{-}F}$ mouse was used as a positive control, green; anti-APP N-terminus antibody (N1D)). $App^{NL\text{-}F}$ mice are an AD model that harbors Swedish and Iberian mutations in the mouse *App* gene and recapitulate amyloid pathology in the brain (Saito et al, Nat Neurosci 2014). A scale bar represents 500 µm. The information on the sex and age of the mice is provided in Supplementary Table 9.

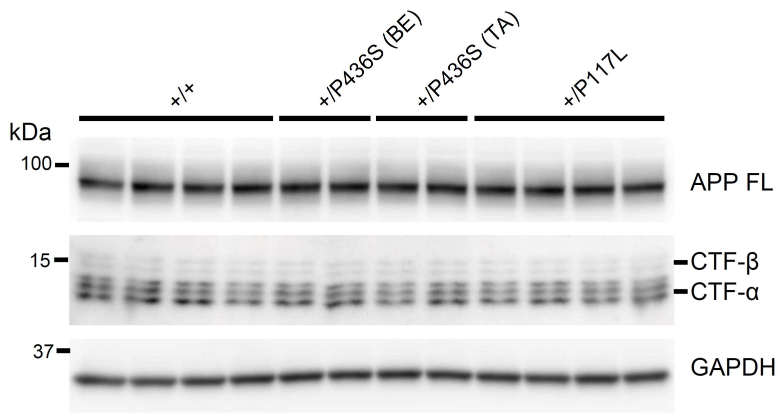

Supplementary figure 6. APP processing in the *Psen1*-P436S and -P117L mouse brains.

Full-length APP (APP FL) and C-terminal fragments (CTF-β and CTF-α) were detected by anti-APP antibody (22C11) or anti APP-CTF antibody. GAPDH was used as loading controls. For *Psen1*-P436S mice, two mice were generated by BE and other two were generated by Target-AID. The information on the sex and age of the mice is provided in Supplementary Table 9.

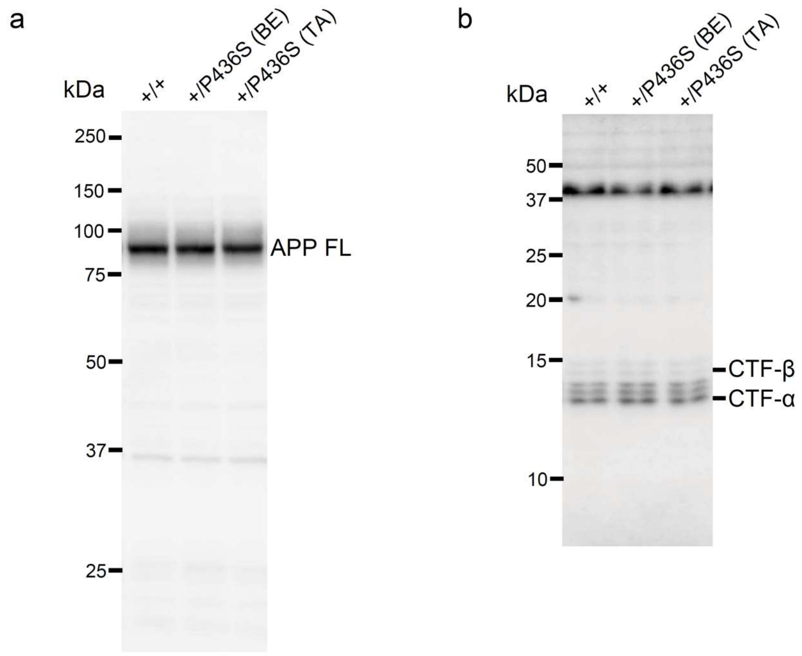

Supplementary figure 7. APP processing in the *Psen1*-P436S mouse brains.

The full-length images of western blots for full-length APP (APP FL) (a) and CTFs (b) in Figure 2b. The information on the sex and age of the mice is provided in Supplementary Table 9.

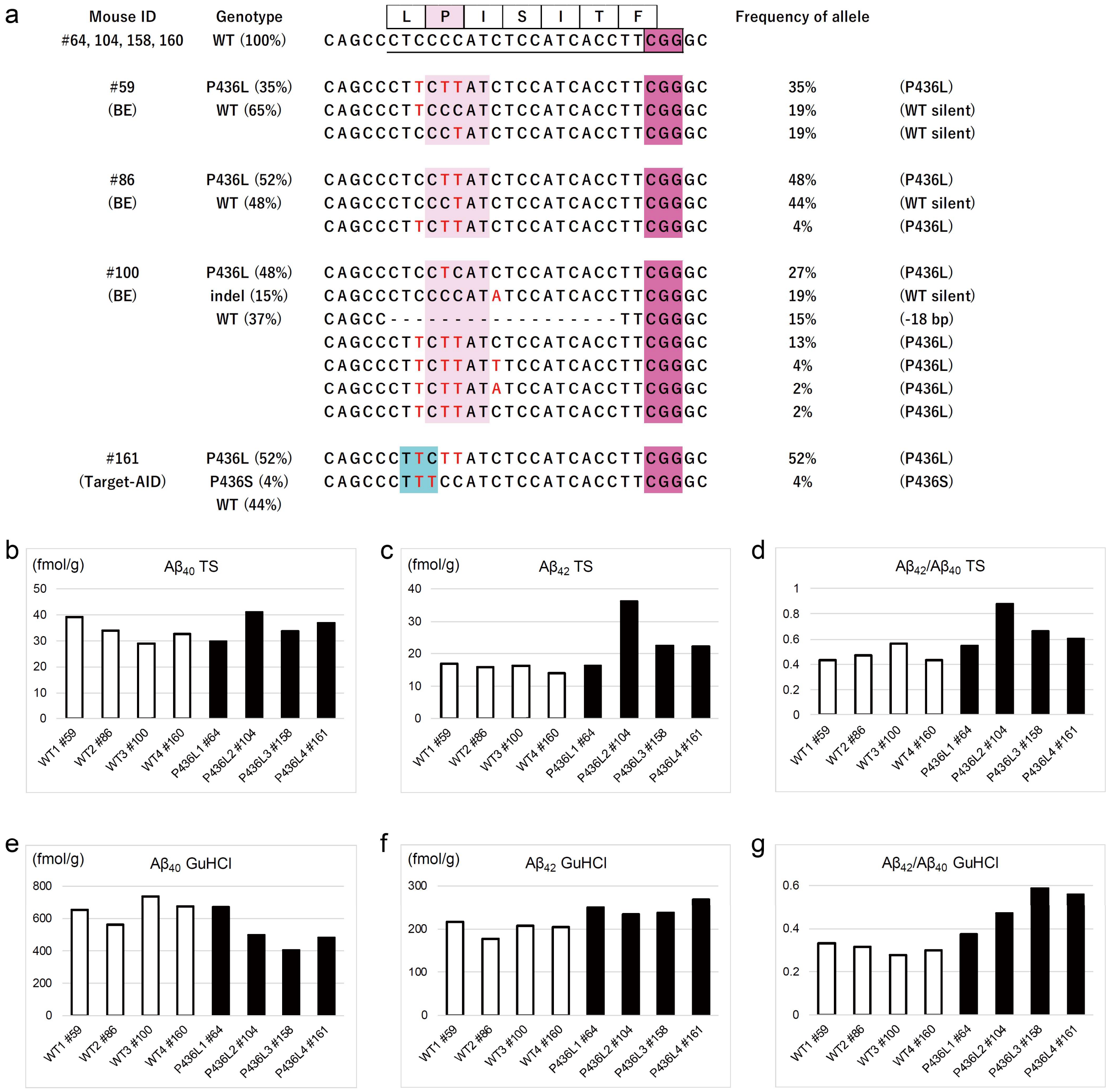

Supplementary figure 8. Characterization of *Psen1*-P436L mice generated by BE or Target-AID.

(a) Alignment of mutant sequences from *Psen1*-P436L founder mice generated by BE (#59, #86, #100) or Target-AID (#161). The sgRNA target sequence is underlined in the wild-type (WT) sequence at the top. The PAM site is shown in magenta, and the active windows of BE and Target-AID are indicated in pink (#59, #86, #100) and light blue (#161), respectively. The substituted bases are indicated in red. The percentages next to the genotype indicate the frequencies of P436L alleles in the left column. The column on the right indicates the frequency of each of the mutant alleles. Note that the mouse #161 had *Psen1*-P436S mutation allele at low frequency (4%) in addition to P436L allele. **(b-g)** Aβ_{40} and Aβ_{42} ELISA in the *Psen1*-P436L mouse brain, performed using brain samples of 3-month-old *Psen1*-P436L mice (F0) generated by BE (#59, #86, #100) or Target-AID

(#161). Cortices from 3-month-old wild-type or heterozygous *Psen1*-P436L mice generated were homogenized and fractionated into Tris-HCl-buffered saline (TS) (**b**-**d**) and guanidine-HCl-soluble (GuHCl) (**e**-**g**) fractions and subjected to the ELISAs for Aβ40 and Aβ42. The information on the sex and age of the mice is provided in Supplementary Table 9.

a

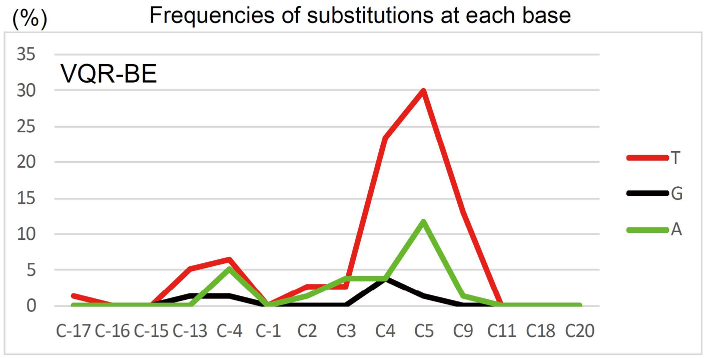

b VQR-BE

Position	-17	-16	-15	-13	-8	-4	-1
Base	C	C	C	C	G	C	C
Substitution	T	-	-	T / G	T / A	T / G / A	-
Frequency (%)	1.3	0	0	5.2/1.3	1.3/1.3	6.5/1.3/5.2	0
Amino acid #	intron	intron	intron	intron	intron	114	115
Amino acid						I > M	Y

1	2	3	4	5	6	7	8	9
A	C	C	C	C	A	T	T	C
-	T / A	T / A	T / G / A	T / G / A	-	-	-	T / A
0	2.6/1.3	2.6/3.9	23.4/3.9/3.9	29.9/1.3/11.7	0	0	0	13.0/1.3
116			117			118		
T > I, N			P > L, S, A, R, Q, I, stop			F > L		

c VQR-BE

Single mutation

P117L*	13 (16.9%)
P117Q	4 (5.2%)
P117S*	3 (3.9%)
P117T	1 (1.3%)
P117V	1 (1.3%)
T116I*	1 (1.3%)
F118L	1 (1.3%)
Total	24/77 (31.2%)

d VQR-BE

Multiple mutations

P117L* / P117S*	5 (6.5%)
P117L* / P117Q	3 (3.9%)
P117S* / P117A*	1 (1.3%)
P117S* / P117Q	1 (1.3%)
P117L* / P117I	1 (1.3%)
T116N* + P117L*	1 (1.3%)
P117L* / P117S* / P117A*	1 (1.3%)
T116N* + P117L* / P117R*	1 (1.3%)
I114M + P117V / P117L*	1 (1.3%)
P117L* / P117T / P117stop	1 (1.3%)
P117S* / P117T / P117V	1 (1.3%)
Totai	17/77 (22.1%)

Supplementary figure 9. Summary of base editing and generation of mutant *Psen1* mice after VQR-BE3 injection targeting *Psen1*-P117.

(**a,b**) Base editing efficiency of VQR-BE at each of the bases in the target site. (**c,d**) Summary of the number of mutant *Psen1* mice obtained after VQR-BE injection. Mutations with asterisks are reported as fAD-related mutations (Alzforum, http://www.alzforum.org). Some mice harbored multiple mutations in same alleles (T116N + P117L and I114M + P117V) or different alleles (P117L/S, P117L/Q, P117S/A, P117S/Q, P117L/I, P117L/S/A, P117L/R, P117L/V, P117L/T/stop, and P117S/T/V).

	DNA sequence	Chromosome	Position	Mismatches		
ON1	ctCCCCATCTCCATCACCTTCGG	chr12	83733969	2	Exon	PSEN1
ON2	ctCCCCATCTCCATCACCTTTGG	chr1	180227651	2	Exon	PSEN2
OFF1	aGCCCCgTCTtCATCACCTTTGG	chr2	15694613	3	Intron	Malrd1
OFF2	aGCCCCAaCTCCAgCACCTTGGG	chr7	27231087	3	Intron	Adck4
OFF3	aGCCCCATCaCCATCAaCTTGGG	chr2	172430998	3	Intron	Cass4
OFF4	aGCCCCATCTCCAaCACCTTGGa	chr7	58968659	3	ncRNA-coding	Gm39020
OFF5	tGCCCCATCTCCcTCcCCTTTGG	chr6	129519253	3	Intergenic	
OFF6	tGCCCCATCTCCATCtCtTTTGG	chr2	4297671	3	Intron	XR_001783625, Frmd4a
OFF7	GaCCCCAcCTCCATaACCTTAGG	chr11	53080597	3	Intron	Fstl4
OFF8	GcCCCCATCcCCATCACCTgTGG	chr15	27850358	3	Intron	Trio
OFF9	GcCCCCATCTCCATCACtTTGGt	chr7	84066461	3	Intron	Cemip
OFF10	GtCCCCATCaCCATCACCTaAGG	chr2	112817876	3	Intron	Ryr3
OFF11	GGaCCCATCTCCcTCAaCTTGGG	chr15	37640429	3	Intron	Ncald
OFF12	GGCaCCATCTCCAgCACCTgTGG	chr17	57047238	3	Exon	Slc25a23
OFF13	GGCtCCtTCTCCATCACCcTGGG	chr4	148000837	3	Exon	Nppa
OFF14	GGCtCCATCTCCAgCACCaTAGG	chr18	10777753	3	Intron	Mib1
OFF15	GGCtCCATCTCCATCtCCTTGGa	chr2	93652051	3	Intron	Alx4
OFF16	GGCtCCATCTCCATCACgcTGGG	chr2	109708716	3	Intron	BDNF
OFF17	GGCtCCATCTCCATCACCaTGGG	chr13	97839690	2	non-coding	
OFF18	GGCCaCATCTgCATCACCTTGGc	chr16	12433236	3	Intergenic	
OFF19	GGCCtCcTaTCCATCACCTTAGG	chr14	10696771	3	Intron	Fhit
OFF20	GGCCtCtTCTCCATCtCCTTGGG	chr6	55133775	3	Intergenic	
OFF21	GGCCCCgTCTCCATCtgCTTTGG	chr14	56402149	3	non-coding	
OFF22	GGCCCCAcCTaCATCACCTcTGG	chr3	88324812	3	Intergenic	
OFF23	GGCCCCAcCTCCATCACCcTTGa	chr7	60108553	3	Intron	Snrpn
OFF24	GGCCCCATgTCCATCcCCTgTGG	chr17	56731055	3	Intergenic	
OFF25	GGCCCCATCTgCcTCACCTaGGG	chr13	31309495	3	non-coding	
OFF26	GGCCCCATCTCCAcCACCTcCtG	chr15	30172157	3	Intergenic	
OFF27	GGCCCCATCTCCAgCACCTgTGG	chr11	87584514	2	Intron	Sept4
OFF28	GGCCCCATCTCCATCACtTgAGG	chr19	24768182	2	Intron	Pgm5
OFF29	GGCCCCATCTCCATCACCccCaG	chr10	78796313	3	Intron	Slc1a6

Supplementary Table 1. Off-target regions in *Psen1*-P436S mice generated by BE3 or Target-AID.
Off-target regions of *Psen1*-P436S mice that accepted up to 3 mismatches were predicted by Cas-OFFinder and COSMID (see methods).

	sgRNA	Injected	Bla/Mor	Abnormal	Indel	Substitution
BE3	mo*App*-Int16-2	60	46	0	16/45 (35.6%)	15/28 (53.6%)
Target-AID		60	50	0	1/48 (2.1%)	14/47 (29.8%)
BE3	mo*App*-Ex17-4	40	25	0	2/25 (8.0%)	8/23 (34.8%)
Target-AID		40	28	1	2/29 (6.9%)	1/29 (3.4%)
BE3	mo*App*-Ex17-3	40	30	1	2/30 (6.7%)	3/30 (10.0%)
Target-AID		40	27	0	1/26 (3.8%)	1/26 (3.8%)

Supplementary Table 2. Summary of the zygotes used after BE or Target-AID injections with sgRNAs targeting the mouse *App* gene.

a Aβ ELISA in 3-month-old *Psen1* -P436S mice (Figure 2c-h)

Mouse ID	*Psen1*	*Psen2*
156004	WT/WT	WT/WT
156014	WT/WT	WT/WT
156015	WT/WT	WT/WT
156017	WT/WT	WT/WT
156018	P436S/WT	WT/WT
156019	P436S/WT	WT/WT
156007	P436S/WT	WT/WT
156021	P436S/WT	WT/WT
89005	P436S/WT	WT/WT
89006	P436S/WT	WT/WT
89007	P436S/WT	WT/WT
89013	P436S/WT	WT/WT

b Aβ ELISA in 3-month-old *Psen1* -P436L mice (Supplementary Fig. 8)

Mouse ID	*Psen1*	*Psen2*
64	WT/WT	WT/WT
104	WT/WT	WT/WT
158	WT/WT	WT/WT
160	WT/WT	WT/WT
59	P436L/WT	WT/WT
86	P436L/WT	WT/WT
100	P436L/WT	WT/WT
161	P436L/P436S/WT	WT/WT

Supplementary Table 3. Genotypes of mutant *Psen1*-P436 mice used in biochemical analysis.
Genotypes of each mice that were used for Aβ ELISA in *Psen1*-P436S mice (a) and *Psen1*-P436L mice (b).

T7-BE3-F	GCCGCTAATACGACTCACTATAG
T7-BE3-R	GCGGGTTTAAACTCAATGGT
T7-Target-AID-F1	GAACCCACTGCTTACTGGCT
T7-Target-AID-R	GTGGAATCGAAATCTCGTGAT

Supplementary Table 4. Primer lists for *in vitro* transcription of BE and Target-AID.

Primers that were used in this experiment are listed. See methods for details of each primer.

sgRNA-moPsen1-P436 (gg18)	taatacgactcactataGGCCCCATCTCCATCACCTTgttttagagctagaa
sgRNA-moPsen1-P117 (gg20)	taatacgactcactataGGACCCCATTCACAGAAGACACgttttagagctagaa
sgRNA-moPsen1-P117 (gg18)	taatacgactcactataGGCCCATTCACAGAAGACACgttttagagctagaa
sgRNA-moApp-Intron 16	taatacgactcactataGGGTCCGCCATCAAAAACTGGTgttttagagctagaa
sgRNA-moApp-Exon 17-1	taatacgactcactataGGCATCATCGGACTCATGGTgttttagagctagaa
sgRNA-moApp-Exon 17-2	taatacgactcactataGGCGCCATCATCGGACTCAgttttagagctagaa
CRISPRscan tail primer	AAAAGCACCGACTCGGTGCCACTTTTTCAAGTTGATAACGGACTAGCCTTATTTTAACTTGCTATTTCTAGCTCTAAAAC

Supplementary Table 5. Primer lists for *in vitro* transcription of sgRNAs.

Primers that were used in this experiment are listed. See methods for details of each primer.

moPS1_gDNA_Ex12_F (P436)	AGAGTATTCCCTGAGCGTGG
moPS1_gDNA_Ex12_R (P436)	ACCGTTCTCTGTAGACGCTT
moPS1_gDNA_Ex5_F (P117)	GGTTGACTGATGACCTGCAA
moPS1_gDNA_Ex5_R (P117)	GCTCAGAACGTGACGCTTAC
moApp_gDNA_Int16_F	ACAGGCATTACATATTCAGCGT
moApp_gDNA_Int16_R	ACTATCAACAGAGCCCCACT
moApp_gDNA_Ex17_F	TGTGCCCTGAGTACCACAGA
moApp_gDNA_Ex17_R	TGCTCATTGTTCCAGAGACG
M13-BDFw	CAGGGTTTTCCCAGTCACGAC
M13-BDRev	CGGATAACAATTTCACACAGG

Supplementary Table 6. Primer lists for sequencing analysis.

Primers that were used in this experiment are listed. See methods for details of each primer.

Supplementary Table 7. Primer lists for targeted deep sequencing analysis (amplicon PCR).

Primers that were used in this experiment are listed. See methods for details of each primer.

ON1-F (Psen1)	TCGTCGGCAGCGTCAGATGTGTATAAGAGACAGTGAGCGTGGTGCTGATGCTG
ON1-R (Psen1)	GTCTCGTGGGCTCGGAGATGTGTATAAGAGACAGCGGGCTTGCTCTCTGTTTTTGTG
ON2-F (Psen2)	TCGTCGGCAGCGTCAGATGTGTATAAGAGACAGCATCCAGTGACGCCCCCATA
ON2-R (Psen2)	GTCTCGTGGGCTCGGAGATGTGTATAAGAGACAGGGCTAGTCTCAGGATTACACCAC
OFF1-F	TCGTCGGCAGCGTCAGATGTGTATAAGAGACAGGGGAATGGTCAGGAAGAACAGAAG
OFF1-R	GTCTCGTGGGCTCGGAGATGTGTATAAGAGACAGGGCTGCCCATTACACTAAGGGA
OFF2-F	TCGTCGGCAGCGTCAGATGTGTATAAGAGACAGAGGGGGCATCAGATCTGGAG
OFF2-R	GTCTCGTGGGCTCGGAGATGTGTATAAGAGACAGGGCCTTTAATCCCAGCACTCAG
OFF3-F	TCGTCGGCAGCGTCAGATGTGTATAAGAGACAGCCACACTGTTTTGTCCCACA
OFF3-R	GTCTCGTGGGCTCGGAGATGTGTATAAGAGACAGTTAATGAGGGGCTGCTGTGA
OFF4-F	TCGTCGGCAGCGTCAGATGTGTATAAGAGACAGAGTCCCCAGAGTTCCAAACATCAC
OFF4-R	GTCTCGTGGGCTCGGAGATGTGTATAAGAGACAGACAGGGGCCCCACATCTTCT
OFF5-F	TCGTCGGCAGCGTCAGATGTGTATAAGAGACAGATGACACCAAGACTTGCCTTCACC
OFF5-R	GTCTCGTGGGCTCGGAGATGTGTATAAGAGACAGAACAGCGGACTCCCTTGCCT
OFF6-F	TCGTCGGCAGCGTCAGATGTGTATAAGAGACAGAGAAAGGAGACCCTCTTAATGGCC
OFF6-R	GTCTCGTGGGCTCGGAGATGTGTATAAGAGACAGTGGCTCCATCAGTGCCTTATGTG
OFF7-F	TCGTCGGCAGCGTCAGATGTGTATAAGAGACAGCCATGCTATTGGGGCCAATTGAG
OFF7-R	GTCTCGTGGGCTCGGAGATGTGTATAAGAGACAGGGACACTTGACACAGCCATGATTC
OFF8-F	TCGTCGGCAGCGTCAGATGTGTATAAGAGACAGAGAAAGCCAGCCCTGGCCTTG
OFF8-R	GTCTCGTGGGCTCGGAGATGTGTATAAGAGACAGGACTCATTACAGGGGTAGAATGGC
OFF9-F	TCGTCGGCAGCGTCAGATGTGTATAAGAGACAGGCTGTGTATAAGCAAGAGACGGC
OFF9-R	GTCTCGTGGGCTCGGAGATGTGTATAAGAGACAGGTGTGGGAGCTGGCACTGTT
OFF10-F	TCGTCGGCAGCGTCAGATGTGTATAAGAGACAGGCTCCTTGCCAGTTATCACACTTC
OFF10-R	GTCTCGTGGGCTCGGAGATGTGTATAAGAGACAGGGGTGCCTTAGCAGACGTAC

OFF11-F	TCGTCGGCAGCGTCAGATGTGTATAAGAGACAGACACACACACACACACACACACACAC
OFF11-R	GTCTCGTGGGCTCGGAGATGTGTATAAGAGACAGGCTTCCCAAAGAGAGCTGTTAGAC
OFF12-F	TCGTCGGCAGCGTCAGATGTGTATAAGAGACAGTACACAGTTACGTGGGAGCAGG
OFF12-R	GTCTCGTGGGCTCGGAGATGTGTATAAGAGACAGGGATCTTCCCGTCTGCAACTC
OFF13-F	TCGTCGGCAGCGTCAGATGTGTATAAGAGACAGACGGGAGATGCTGGCAGCTA
OFF13-R	GTCTCGTGGGCTCGGAGATGTGTATAAGAGACAGAGACCCTGGTCCCAGTCTGT
OFF14-F	TCGTCGGCAGCGTCAGATGTGTATAAGAGACAGGACCCTCCTGCCTCTTTTTTCTG
OFF14-R	GTCTCGTGGGCTCGGAGATGTGTATAAGAGACAGGGCCTACAAGTACTGCCATAGGT
OFF15-F	TCGTCGGCAGCGTCAGATGTGTATAAGAGACAGAGGCTTGAGATACACAGTAGGTGC
OFF15-R	GTCTCGTGGGCTCGGAGATGTGTATAAGAGACAGGTTGGGGATAGATGGATCTCCTC
OFF16-F	TCGTCGGCAGCGTCAGATGTGTATAAGAGACAGCAACTCAACAGTTAACTCTGACTAACTATGAGC
OFF16-R	GTCTCGTGGGCTCGGAGATGTGTATAAGAGACAGCATGTTTCAGAAACCCTCATATAGGATTTTGCC
OFF17-F	TCGTCGGCAGCGTCAGATGTGTATAAGAGACAGTCGGTGAAAGGGGAGAGAGC
OFF17-R	GTCTCGTGGGCTCGGAGATGTGTATAAGAGACAGGAGCTCTTACACAGGAGTGTTGTC
OFF18-F	TCGTCGGCAGCGTCAGATGTGTATAAGAGACAGCTTCCATCTACGTCTTCCAGTCTC
OFF18-R	GTCTCGTGGGCTCGGAGATGTGTATAAGAGACAGTCTCTCAACTCCCCTCCCCA
OFF19-F	TCGTCGGCAGCGTCAGATGTGTATAAGAGACAGATCACAGGACCGTAGCTCTC
OFF19-R	GTCTCGTGGGCTCGGAGATGTGTATAAGAGACAGTATAGATCAGGCTCGCTGGG
OFF20-F	TCGTCGGCAGCGTCAGATGTGTATAAGAGACAGTTAAGAGGGTCAGGCTTTTCCTGG
OFF20-R	GTCTCGTGGGCTCGGAGATGTGTATAAGAGACAGTCCCACTGAGGCTCTGAGATTG
OFF21-F	TCGTCGGCAGCGTCAGATGTGTATAAGAGACAGAGTCTTACCCAGGAGCCACC
OFF21-R	GTCTCGTGGGCTCGGAGATGTGTATAAGAGACAGCTTGTAGATGAGGCTCAGGAGCT
OFF22-F	TCGTCGGCAGCGTCAGATGTGTATAAGAGACAGAAGTTGGAATAGCCTCCCGAAAGC
OFF22-R	GTCTCGTGGGCTCGGAGATGTGTATAAGAGACAGCCTTCGGAAAAGCAGTCAGTGCTC
OFF23-F	TCGTCGGCAGCGTCAGATGTGTATAAGAGACAGGGAGCCAGTGTGGAGCAAGT
OFF23-R	GTCTCGTGGGCTCGGAGATGTGTATAAGAGACAGGTTCTGGGGTGGGTGAGTATG
OFF24-F	TCGTCGGCAGCGTCAGATGTGTATAAGAGACAGCCTGTGTACCTGTGGCTTCT
OFF24-R	GTCTCGTGGGCTCGGAGATGTGTATAAGAGACAGTCTGATGAGGGTCTTGGCTC

OFF25-F	TCGTCGGCAGCGTCAGATGTGTATAAGAGACAGCACTTCACGCAGGAGATGGG
OFF25-R	GTCTCGTGGGCTCGGAGATGTGTATAAGAGACAGGGAATCTGTGATTTCCCTCCACC
OFF26-F	TCGTCGGCAGCGTCAGATGTGTATAAGAGACAGGAGTTAAGGAGAGCACCGGG
OFF26-R	GTCTCGTGGGCTCGGAGATGTGTATAAGAGACAGGTGAGACGTGACAGGAAGACGA
OFF27-F	TCGTCGGCAGCGTCAGATGTGTATAAGAGACAGTCATCCACCAGGAGAATCTGGTC
OFF27-R	GTCTCGTGGGCTCGGAGATGTGTATAAGAGACAGACCACACCCAGCACAGCATC
OFF28-F	TCGTCGGCAGCGTCAGATGTGTATAAGAGACAGCCCTCATTGGCCAGCTGATC
OFF28-R	GTCTCGTGGGCTCGGAGATGTGTATAAGAGACAGCCTCTTCCTGCCCCTTAAATAGC
OFF29-F	TCGTCGGCAGCGTCAGATGTGTATAAGAGACAGGGGACTTCTTTGACAGCCTCAATG
OFF29-R	GTCTCGTGGGCTCGGAGATGTGTATAAGAGACAGGAGCATAGACAGATGAGTGGGTG

i7_Primer_Fw	CAAGCAGAAGACGGCATACGAGATGTAGCTCCGTCTCGTGGGCTCGG
i5_Primer_Rev_WT	AATGATACGGCGACCACCGAGATCTACACCTCTCTATTCGTCGGCAGCGTC
i5_Primer_Rev_P436S_BE	AATGATACGGCGACCACCGAGATCTACACTATCCTCTTCGTCGGCAGCGTC
i5_Primer_Rev_P436S_TA	AATGATACGGCGACCACCGAGATCTACACGTAAGGAGTCGTCGGCAGCGTC

Supplementary Table 8. Primer lists for targeted deep sequencing analysis (index PCR).

Primers that were used in this experiment are listed. See methods for details of each primer.

Figure number	Assays	Genotype	Sex	Age
Figure 2b	Western blot	+/+ (WT)	Female	3-month-old
		+/P436S (BE)	Female	3-month-old
		+/P436S (TA)	Female	3-month-old
Figure 2c-h	ELISA	+/+	Female	3-month-old
		+/P436S (BE)	Female	3-month-old
		+/P436S (TA)	Female	3-month-old
Figure 3d-i	ELISA	+/+	Female	3-month-old
		+/P117L	Female	3-month-old
Supplementary Figure 5a-b	picture and Western blot	+/+	Unknown	Postnatal day 5
		+/P436S	Unknown	Postnatal day 5
		P436S/P436S	Unknown	Postnatal day 5
Supplementary Figure 5c-f	ELISA	+/P436S	Female (n=2), Male (n=1)	6-week-old
		+/P436S	Female (n=8)	3-month-old
		+/P436S	Male (n=3)	9-month-old
Supplementary Figure 5g	IHC	$App^{NL\text{-}F}$	Male	24-month-old
		+/+	Male	9-month-old
		+/P436S	Male	9-month-old
Supplementary Figure 6	Western blot	+/+	Female	3-month-old
		+/P436S (BE)	Female	3-month-old
		+/P436S (TA)	Female	3-month-old
		+/P117L	Female	3-month-old
Supplementary Figure 7	Western blot	+/+ (WT)	Female	3-month-old
		+/P436S (BE)	Female	3-month-old
		+/P436S (TA)	Female	3-month-old
Supplementary Figure 8	ELISA	WT	Female (n=1), Male (n=3)	3-month-old
		P436L	Female	3-month-old

Supplementary Table 9. Age and sex of mice used in biochemical analyses.
